# Systematic review and meta-analysis of laparoscopic and open gastrectomy for advanced gastric cancer

**DOI:** 10.1186/1477-7819-11-182

**Published:** 2013-08-08

**Authors:** Ke Chen, Xiao-Wu Xu, Yi-Ping Mou, Yu Pan, Yu-Cheng Zhou, Ren-Chao Zhang, Di Wu

**Affiliations:** 1 Department of General Surgery, Sir Run Run Shaw Hospital, School of Medicine, Zhejiang University, 3 East Qingchun Road, Hangzhou, Zhejiang Province 310016, China

## Abstract

**Background:**

The use of laparoscopic gastrectomy (LG) in advanced gastric cancer (AGC) remains a controversial topic, mainly because of doubts about its oncologic validity. This study is a systematic review and meta-analysis of the available evidence.

**Methods:**

A comprehensive search was performed until June 2013 to identify comparative studies evaluating survival rates, recurrence rates, surgical outcomes and complications. Pooled risk ratios (RR) and weighted mean differences (WMD) with 95% confidence intervals (CI) were calculated using the random effects model. Data synthesis and statistical analysis were carried out using RevMan 5.1 software.

**Results:**

Fifteen trials were involved in this analysis. Compared to open gastrectomy (OG), LG involved a longer operating time (WMD = 48.67 min, 95% CI 34.09 to 63.26, *P* < 0.001); less blood loss (WMD = −139.01 ml, 95% CI −174.57 to −103.44, *P* < 0.001); earlier time to flatus (WMD = −0.79 days, 95% CI −1.14 to −0.44, *P* < 0.001); shorter hospital stay (WMD = −3.11 days, 95% CI −4.13 to −2.09, *P* < 0.001); and a decrease in complications (RR = 0.74, 95% CI 0.61 to 0.90, *P* = 0.003). There was no significant difference in the number of harvested lymph nodes, margin distance, mortality, cancer recurrence rate and long-term survival rate between the AGC patients treated with LG or OG (*P* > 0.05).

**Conclusions:**

Despite a longer operation, LG is a safe technical alternative to OG for AGC with a lower complication rate and enhanced postoperative recovery. Moreover, there were similar outcomes between both approaches in terms of cancer recurrence and the long-term survival rate. Because of the limitation of this study, methodologically high-quality studies are needed for further evaluation.

## Background

Although the annual incidence of and mortality from gastric cancer have been decreasing yearly worldwide, gastric cancer still accounts for more than 10% of cancer deaths worldwide and is the second most frequent cause of cancer death after lung cancer [1,2]. Adjuvant chemotherapy improves the survival of these patients [3,4], but radical gastrectomy with regional lymph node dissection still remains the only potentially curative treatment available for gastric adenocarcinoma [5,6].

Since the first report of laparoscopic gastrectomy (LG) for early gastric cancer (EGC) by Kitano [7], it has undergone rapid development and gained popularity in the past 20 years. Compared to traditional open gastrectomy (OG), most studies have reported that LG can achieve better cosmesis, shorter hospital stay, faster recovery and better postoperative quality of life [8-13]. However, most of these studies focus on EGC. LG for advanced gastric cancer (AGC) remains controversial and has not achieved universal acceptance because of its uncertain oncological safety, particularly given the technical difficulty of lymphadenectomy for metastatic lymph nodes [14]. Meanwhile, there have been few long-term follow-up results regarding the oncological adequacy of laparoscopic surgery compared to that of open surgery for AGC.

Although several meta-analyses and systematic reviews have demonstrated the safety and oncological effect of LG for EGC [15-19], such studies have not been conducted for the potential benefits and disadvantages of LG for AGC. The aim of this study was to compare LG with OG with respect to morbidity, mortality, intraoperative outcomes and functional recovery. Long-term outcomes after LG and OG in patients with AGC were evaluated in a systematic review of the literature, and meta-analyses were performed.

## Methods

### Literature search

A systematic search using the following keywords, “laparoscopy”, “laparoscopic”, “gastric cancer”, “gastric carcinoma” and “gastrectomy”, was performed through the following bibliographic databases, PubMed, Web of Science and Cochrane Library, for literature comparing LG and OG published between January 1995 and June 2013, and we broadened the search range by browsing the related summary, methods and references of retrieved articles. The language of the publications was confined to English. Two investigators reviewed the titles and abstracts, and assessed the full text to establish eligibility.

### Study selection criteria

All clinical studies needed to meet the following criteria for the meta-analysis: (1) being published in English with data comparing LG and OG for AGC; (2) having clear case selection criteria and surgical methods; they had to contain long-term outcomes such as tumor recurrence and survival rate; (3) articles referring only or predominantly to AGC, because it is difficult to confirm AGC preoperatively [20]. However, articles with significant differences in tumor stages between groups were excluded. (4) If there was an overlap between authors or centers, only the higher quality or more recent literature was selected. However, articles from the same authors or centers but with different patient cohorts were included.

### Data extraction and quality assessment

Two authors independently extracted the data using a unified data sheet and decided upon the controversial issues through discussion. Extracted data included the author, study period, geographical region, number of patients, operating time, blood loss, number of retrieved lymph nodes, proximal and distal margin distance, time to flatus, time to oral intake, length of hospital stay, morbidity and mortality, tumor recurrence and survival rate. Postoperative complications were classified as medical (cardiovascular, respiratory or metabolic events; nonsurgical infections; deep venous thrombosis; pulmonary embolism) or surgical (any anastomotic leakage or fistula, any complication that required reoperation, intra-abdominal collections, wound complications, bleeding events, pancreatitis, ileus, delayed gastric emptying and anastomotic stricture). This classification system is based on the Memorial Sloan-Kettering Cancer Center complication reporting system [21]. If necessary, the first authors were contacted to retrieve further information.

Randomized controlled trials (RCTs) were evaluated by the Jadad composite scale. High-quality trials scored more than 2 out of a maximum possible score of 5. The Newcastle-Ottawa Quality Assessment Scale (NOS) was used for quality assessment of observational studies. A threshold of six stars or above has been considered indicative of high quality.

### Statistical analysis

The meta-analysis was performed in line with recommendations from the Cochrane Collaboration and the Quality of Reporting of Meta-Analyses guidelines [22,23]. Continuous variables were assessed using the weighted mean difference (WMD), and dichotomous variables were analyzed using the risk ratio (RR). If the study provided medians and ranges instead of means and standard deviations (SDs), we estimated the means and SDs as described by Hozo *et al*. [24]. To account for clinical heterogeneity, which refers to diversity in a sense that is relevant for clinical situations, we used the random effects model based on DerSimonian and Laird’s method. Potential publication bias was determined by conducting informal visual inspection of funnel plots based on the complications. Data analyses were performed using Review Manage version 5.1 (RevMan 5.1) software downloaded from the Cochrane Library. *P* < 0.05 was considered statistically significant.

## Results

### Studies selected

**Figure 1:**
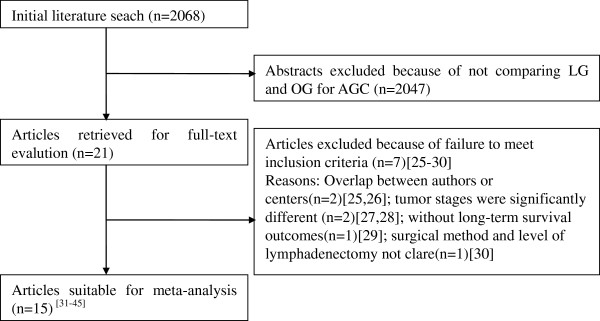
Flow chart of literature search strategies.

The initial search strategy retrieved 2,068 publications in English. After the titles and abstracts had been reviewed, papers without comparison of LG and OG for AGC were excluded, which left 21 comparative studies, 6 [25-30] of which did not meet the inclusion criteria and were excluded. This left a total of two RCTs and 13 observational studies [31-45], all of which were accessible in full-text format. A flow chart of the search strategies is illustrated in Figure 1.

### Study characteristics and quality

A total of 2,519 patients were included in the analysis with 1,327 undergoing LG (52.7%) and 1,192 undergoing OG (47.3%). They represent international experience including data from five different countries (six from China, four from Korea, three from Italy, one from Japan and one from Chile). Two RCTs got Jadad scores of 2 and 3, respectively. According to the NOS, 2 out of the 13 observational studies got 7 stars, 6 articles got 8 stars, and the remaining 5 got 9 stars. The characteristics and methodological quality assessment scores of the included studies are shown in Table 1.

**Table 1:** Characteristics of included studies

**Author**	**Country**	**Study type**	**Publication year**	**Study period**	**Sample size**		**Level of lymphadenectomy**	**Surgical extension**	**Reconstruction**	**Adjuvant chemotherapy (%)**		**Quality scores**
					**LG**	**OG**				**LG**	**OG**	
Huscher	Italy	RCT	2005	1992-1996	30	29	D1, D2	DG	B-II, R-Y	NR	NR	3*
Hur	Korea	Retrospective	2008	2004-2007	26	25	D2	DG	B-I, B-II	76.9	68	8
Du XH	China	Retrospective	2009	2004-2008	78	90	D2	DG	B-I, B-II	NR	NR	7
Hwang	Korea	Retrospective	2009	2004-2007	45	83	D1 + *α/*β, D2	DG	B-I, B-II	93.2	89.0	7
Du J	China	Retrospective	2010	2005-2009	82	94	D2	TG	R-Y	100	100	8
Cai	China	RCT	2011	2008-2009	49	47	D2	DG, PG, TG	B-I, B-II, R-Y	100	100	2*
Scatizzi	Italy	Retrospective	2011	2006-2009	30	30	D2	DG	R-Y	NR	NR	8
Shuang	China	Retrospective	2011	2005-2007	35	35	D2	DG	B-II	NR	NR	8
Zhao	China	Retrospective	2011	2004-2009	346	313	D1 + *α*/β, D2	DG	B-I, B-II	93.1	91.7	9
Chen	China	Retrospective	2012	2008-2010	224	112	D2	DG, TG	B-I, B-II, R-Y	NR	NR	8
Chun	Korea	Retrospective	2012	2004-2009	52	67	D2	DG	B-I, B-II, R-Y	NR	NR	9
Kim	Korea	Retrospective	2012	1999-2007	88	88	D2	DG, TG	B-I, B-II, R-Y	NR	NR	9
Moisan	Chile	Retrospective	2012	2005-2010	31	31	D1 + α/β, D2	DG, TG	B-II, R-Y	19.4	22.6	8
Siani	Italy	Retrospective	2012	2003-2009	25	25	D1 + α/β, D2	TG	R-Y	NR	NR	9
Shinohara	Japan	Retrospective	2013	1998-2008	186	123	D2	DG, PG, TG	B-I, R-Y	61.3	58.5	9

Abbreviations: *RCT* Randomized controlled trial, *LG* Laparoscopic gastrectomy, *OG* Open gastrectomy, *DG* Distal gastrectomy, *PG* Proximal gastrectomy, *TG* Total gastrectomy, *B*-*I* Billroth-I, *B*-*II* Billroth-II, *R*-*Y* Roux-en-Y, *NR* Not reported. *Jadad scores.

### Intraoperative effects

**Figure 2:**
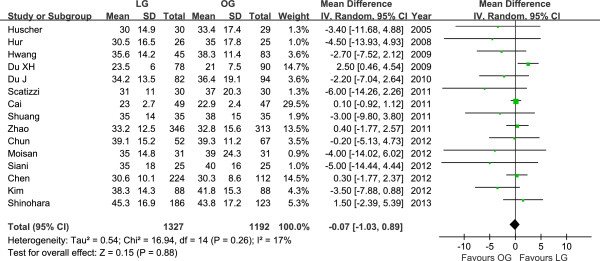
Meta-analysis of the pooled data: number of retrieved lymph nodes.

The mean operating time of LG was 48.67 min longer than for OG (WMD = 48.67 min, 95% CI 34.09 to 63.26, *P* < 0.001). The intraoperative blood loss was lower in LG than in OG (WMD = −139.01 ml, 95% CI −174.57 to −103.44, *P* < 0.001). All studies contained the number of retrieved lymph nodes. The difference in the mean number of retrieved lymph nodes between LG and OG was not significant in the pooled data (WMD = −0.07, 95% CI −1.03 to 0.89, *P* = 0.88) (Figure 2). Meta-analysis of the distal margin distance showed no significant difference between the two groups (WMD = 0.08 cm, 95% CI −0.16 to 0.32, *P* = 0.50). However, the proximal margin distance of OG was longer than that of LG with a marginal difference (WMD = −0.26 cm, 95% CI −0.54 to 0.01, *P* = 0.06). All intraoperative effect outcomes are summarized in Table 2.

**Table 2:** Pooled short-term outcomes of meta-analysis

**Outcomes**	**No. of studies**	**Sample size**		**Heterogeneity ( *P *, *I***^***2***^**)**	**Overall effect size**	**95% CI of overall effect**	***P *value**
		**LG**	**OG**				
Operating time (min)	15	1327	1192	<0.001, 93%	WMD = 48.67	34.09−63.06	<0.001
Blood loss (ml)	12	1157	1007	<0.001, 91%	WMD = −139.01	−174.57− −103.44	<0.001
Retrieved lymph nodes	15	1327	1192	0.26, 17%	WMD = −0.07	−1.03−0.89	0.88
Proximal margin (cm)	6	588	607	0.27, 21%	WMD = −0.26	−0.54−0.01	0.06
Distal margin (cm)	4	517	499	0.86, 0%	WMD = 0.08	−0.16−0.32	0.50
Analgesics given (days)	4	192	242	<0.001, 89%	WMD = −1.57	−2.40− −0.74	<0.001
Time to ambulation (days)	6	913	755	<0.001, 96%	WMD = −1.01	−1.56− −0.45	<0.001
Time to first flatus (days)	11	1045	974	<0.001, 94%	WMD = −0.79	−1.14− −0.44	<0.001
Time to oral intake (days)	9	967	793	<0.001, 87%	WMD = −1.06	−1.63− −0.50	<0.001
Hospital stay (days)	14	1238	1091	<0.001, 84%	WMD = −3.11	−4.13− −2.09	<0.001
Overall complications	15	1327	1192	0.66, 0%	RR = 0.74	0.61−0.90	0.003
Surgical complications	15	1,327	1,192	0.56, 0%	RR = 0.73	0.58−0.92	0.007
Medical complications	12	868	754	0.40, 5%	RR = 0.65	0.41−1.02	0.06
Mortality	7	965	821	0.75, 0%	RR = 0.78	0.30−2.04	0.61

Abbreviations: *WMD* Weighted mean difference, *RR* Risk ratio.

### Postoperative outcome

The mean time to first flatus was shorter in LG than in OG (WMD = −0.79 d, 95% CI −1.14 to −0.44, *P* < 0.001), as was the time to restart oral intake after surgery (WMD = −1.06 d, 95% CI −1.63 to −0.50, *P* < 0.001). A shorter hospital stay was also observed in the LG group (WMD = −3.11 d, 95% CI −4.13 to −2.09, *P* < 0.001).

**Figure 3:**
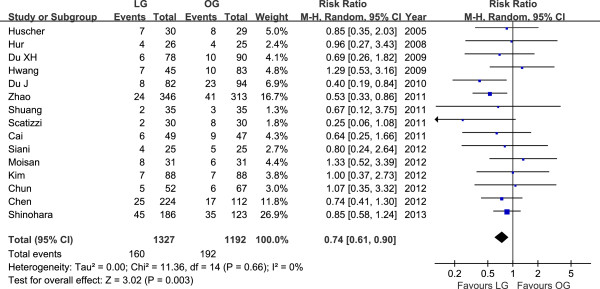
Meta-analysis of the pooled data: overall postoperative complications.

**Figure 4:**
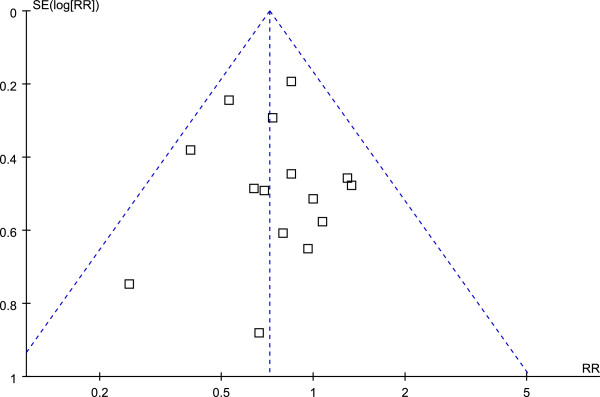
Funnel plot of the overall postoperative complications.

Mortality was described in seven studies, and there was no significant difference in postoperative mortality (RR = 0.78, 95% CI 0.30 to 2.04, *P* = 0.61). The rate of overall postoperative complications was lower for LG (RR = 0.74, 95% CI 0.61 to 0.90, *P* = 0.003) (Figure 3). Visual inspection of the funnel plot revealed symmetry, indicating no serious publication bias (Figure 4). After further analysis, surgical complications were also lower for LG (RR = 0.73, 95% CI 0.58 to 0.92, *P* = 0.007). In analyzing the specific complications, wound infection and ileus were lower for LG (wound infection: RR = 0.53, 95% CI 0.33 to 0.85, *P* = 0.009; ileus: RR = 0.27, 95% CI 0.09 to 0.75, *P* = 0.01). Other surgical complications such as anastomotic leakage, intra-abdominal collections, bleeding or anastomotic stricture were similar between groups (*P* > 0.05). Besides, LG was associated with a marginal reduction in medical complications (RR = 0.65, 95% CI 0.41 to 1.02, *P* = 0.06) with a possible contribution from respiratory complications (RR = 0.57, 95% CI 0.30 to 1.10, *P* = 0.09). All postoperative outcomes are summarized in Table 2.

### Recurrence and long-term survival rate

**Figure 5:**
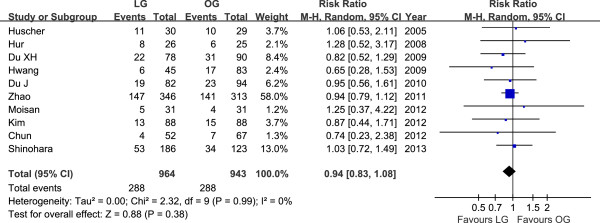
Meta-analysis of the pooled data: recurrences.

Ten studies reported the cancer recurrence [31-35,39,41-43,45]. The recurrence risk in LG was 29.9% (288/964) and 30.5% (288/943) in OG, but the difference between LG and OG was not significant (RR = 0.94, 95% CI 0.83 to 1.08, *P* = 0.38) (Figure 5). The available data about recurrence patterns and specific recurrent sites are summarized in Table 3.

**Table 3:** Systematic review of recurrence patterns and specific recurrent sites

**Author**	**Group**	**Sample size**	**Tumor recurrence**						
			**Total**	**LR**	**LN**	**Liver**	**Lung**	**Bone**	**Peritoneum**
Hur	LG	26	8	3	1	1		2	1
	OG	25	6		5	1			
Du J	LG	82	19	10		5	4		
	OG	94	23	10		7	4	2	
Chun*	LG	52	4		3	2		1	
	OG	67	7	1	5	1		1	3
Kim*	LG	88	13	2	4	6		3	5
	OG	88	15	2	4	4	3		2

Abbreviations: *LR* Local recurrence, *LN* Lymph node; *some patients had mixed tumor recurrence.

Another four studies [31,43-45] available reported no port-site metastases in the LG group. Hwang *et al*. [34] reported a port-site recurrence 10 months after LG. Zhao *et al*. [39] reported a case of port-site recurrence 13 months after LG group; a case of incision metastasis and a case of metastasis in the orifice of the abdominal drain tube 27 and 9 months, respectively, after OG group. Moison *et al*. [43] reported tumors recurred in distant sites in three patients in the LG and in two patients in the OG group, and a recurrence in the remnant stomach in the LG group. Shinohara *et al*. [45] reported 53 recurrences in the LG group: 29 (54.7%) from peritoneal recurrence, 23 (43.4%) from distant or hematogenous recurrence and 15 (28.3%) from locoregional or lymphatic recurrence; the corresponding findings in the OG group were 17 (50%), 15 (44.1%) and 11 (32.6%), respectively.

**Figure 6:**
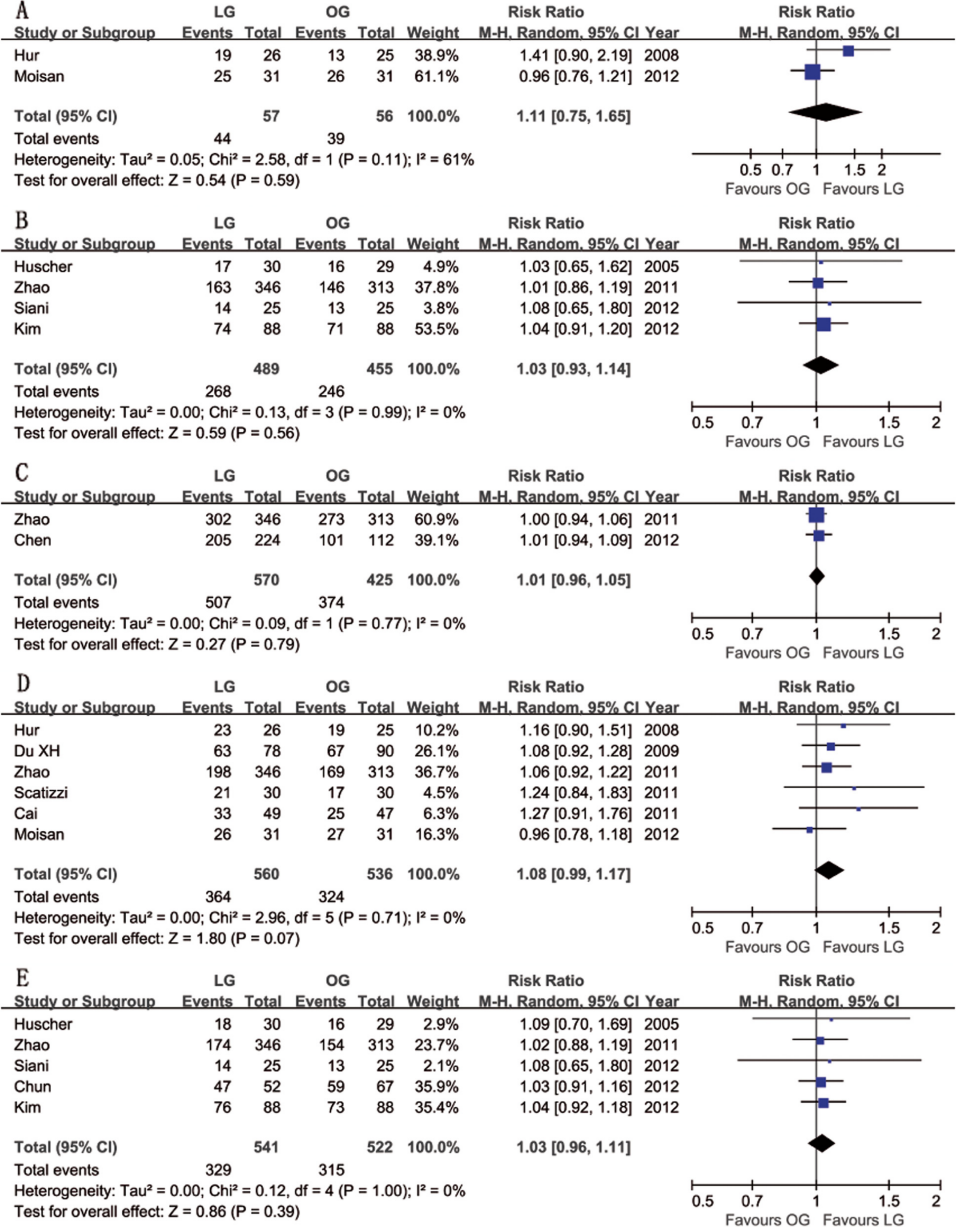
**Meta-analysis of the pooled data: survival rate. (A)** 3y-DFS, **(B)** 5y-DFS, **(C)** 1y-OS, **(D)** 3y-OS and **(E)** 5y-OS. *DFS*, disease-free survival; *OS*, overall survival; *y*, year.

Twelve studies reported postoperative survival rates [31-33,36,37,39-45], all of which did not find significant differences in survival rates between groups. Although Shuang *et al*. [38] did not report specific survival rates, they also found no significant difference in the survival rates between the two groups after 50 months of follow-up (*P* > 0.05). Meta-analysis of these available data demonstrated that the disease-free survival (DFS) rate was not significantly different in participants who received LG compared with OG (3-year: RR = 1.11, 95% CI 0.75 to 1.65, *P* = 0.59; 5-year: RR = 1.03, 95% CI 0.93 to 1.14, *P* = 0.56) (Figure 6), nor was the overall survival (OS) rate (1-year: RR = 1.01, 95% CI 0.96 to 1.05, *P* = 0.79; 3-year: RR = 1.08, 95% CI 0.99 to 1.17, *P* = 0.07; 5-year: RR = 1.03, 95% CI 0.96 to 1.11, *P* = 0.39) (Figure 6). The systematic review outcomes of long-term survival rates are summarized in Table 4.

**Table 4:** Systematic review of long-term survival outcomes

**Author**	**Group**	**Follow-up (months)**	**Recurrence**	**DFS rate**	**OS rate**
Huscher	LG	60 (2–88)	11	5-y: 57.3	5-y: 58.9
	OG	55 (7–90)	10	5-y: 54.8	5-y: 55.7
Hur	LG	29 (6–47)	8	3-y: 71.4	3-y: 88.2
	OG		6	3-y: 53.4	3-y: 77.2
Du XH	LG	25.2 (4–58)	22	NR	3-y: 81.2
	OG		31	NR	3-y: 74.7
Hwang	LG	23 (9–40)	6	NR	NR
	OG	23.5 (8–41)	17	NR	NR
Du J	LAG	25 (2–44)	19	NR	NR
	OG		23	NR	NR
Cai	LG	22.1 (4–36)	NR	NR	3-y: 67.1
	OG		NR	NR	3-y: 53.8
Scatizzi	LG	18 (2–37)	NR	NR	3-y: 70.9
	OG	18 (7–42)	NR	NR	3-y: 56.8
Shuang	LG	36.5 (23–50)	NR	NR	NR
	OG	38.5 (27–50)	NR	NR	NR
Zhao	LG	37 (6–72)	147	5-y: 47	1-y: 87.2, 3-y: 57.2, 5-y: 50.3
	OG		141	5-y: 46.8	1-y: 87.1, 3-y: 54.1, 5-y: 49.2
Chen	LG	19 (1–48)	NR	NR	1-y: 91.5
	OG		NR	NR	1-y: 89.8
Chun	LG	53.2 (1–82.2)	4	NR	5-y: 91.3
	OG	60.4 (7–91.7)	7	NR	5-y: 88.6
Kim	LG	53.7 (8.3−138.1)	13	5-y: 84.6	5-y: 85.9
	OG	58.1 (0.3−106.2)	15	5-y: 81.1	5-y: 83.1
Moisan	LG	28	5	3-y: 79.4	3-y: 82.3
	OG	40	4	3-y: 83.4	3-y: 86.9
Siani	LG	32.6	NR	5-y: 54.2	5-y: 55.7
	OG	31.9	NR	5-y: 52.1	5-y: 52.9
Shinohara	LG	48.8 (25–58.5)^a^	53	5-y: 65.8^b^	5-y: 68.1^b^
	OG		34	5-y: 62.0^b^	5-y: 63.7^b^

Abbreviations: *DFS* Disease-free survival, *OS* Overall survival, *y* Year, *NR* Not reported.

Follow-up time are shown as median (range); ^a^shown as interquartile range; ^b^calculated by excluding stage IA and missing follow-up patients.

## Discussion

RCTs are the most ideal tool for meta-analysis. However, it is difficult to conduct a high-quality RCT to evaluate a new surgical intervention because of obstacles such as learning curve effects, ethical and cultural resistance, and urgent or unexpected conditions during the operation. For these reasons, to include non-RCTs is an appropriate strategy to extend the source of evidence. Therefore, our meta-analysis synthesized the existing observational studies with strictly limiting inclusion and exclusion criteria to evaluate the safety and efficacy of LG in patients with AGC to determine whether LG is an acceptable alternative to OG. The quality scores of the included observational studies got 7 or more stars according to the NOS. These studies were primarily derived from the countries with the most widespread use of LG and mainly published in the past 5 years (2009–2013). Meta-analysis conducted based on this principle will contribute a more comprehensive and objective evaluation for the current status of LG treating AGC.

Reduction in the intraoperative blood loss is a consistent finding in studies comparing laparoscopic and open techniques in many different clinical situations. This is because laparoscopic surgery is more delicate than open surgery in providing perfect amplification. Regarding the operating time, LG is more time-consuming than OG. LG combined with lymphadenectomy is a complex operation and needs extensive technical expertise. Studies designed to estimate the learning curve have shown a significant reduction in operating time after about 50 LG cases [46-48]. Research from some large specialized centers reported that the operating time of LG was not longer than OG in experienced hands [40,49]. Various modified techniques could help to simplify the procedure of reconstruction and shorten the operating time [49,50]. Therefore, researchers expect that with proficiency in the laparoscopic technique and continuous improvement of equipment the time required for LG will become shorter [17].

One of the most striking findings was a reduced number of complications including surgical and medical ones in the LG versus OG group. Meta-analysis of the specified complications demonstrated that wound infections and ileus were significantly less common in the LG group. The reduced surface area of incisions and the manual handling of organs limit the risk of surgical site infections and ileus. It was not surprising that other surgical complications were not reduced because the laparoscopic technique, although less invasive, results in the same organ and lymphatic resection as the open procedure. Besides, the decreased medical complications could be explained by the reduced invasiveness of the laparoscopic technique and less pain after surgery. We also found that respiratory complications occurred in LG less often than in OG, although the difference was not significant (*P* = 0.09). The pain caused by a large incision as well as the use of tension sutures and abdominal bandages after laparotomy can make it difficult for patients to cough, expectorate and perform breathing exercises effectively, thus leading to such complications as pulmonary infection [51]. Reduced use of analgesic drugs, shortened time of abdominal cavity exposure and earlier postoperative activities are considered to be the main reasons for earlier gastrointestinal recovery from LG.

The concern about the technical difficulty of lymphadenectomy for perigastric lymph nodes is one of the major obstacles to accepting LG for AGC. Indeed, the adequacy of the radical resection should be evaluated by the extent of lymph node dissection performed and the number of retrieved lymph nodes. Our meta-analysis -revealed that there was no evident difference in the number of lymph nodes dissected between two groups, which was different from the results of some early meta-analyses [16,17,52]. In recent years, with improved equipment and increased surgeon experience, the number of lymph nodes dissected by LG has gradually increased [8,53]. Moreover, some researchers have reported not only a similar number of overall retrieved lymph nodes between LG and OG, but also a similar number of specific lymph nodes, such as group 7, 8a, 9, 11p, 12a and 14v, which used to be considered difficult for laparoscopic dissection [54]. Park *et al*. [55] evaluated the long-term outcomes of 239 patients who underwent LG for the treatment of advanced gastric cancer. They found that the major recurrence was distant metastasis, whereas relapsed lymph nodes were most frequent in para-aortic or distant lymph node metastasis. Therefore, we believe that the dissection of lymph nodes around the stomach can be performed efficiently under laparoscope. Besides, splenic hilar lymph node dissection is one of the difficulties in upper and middle gastric cancer because the splenic vessels run circuitously, the branches vary substantially, and they are in a narrow and deep space. Therefore, it is easy to cause hemorrhage or spleen ischemia and further necrosis accidentally. Compared to laparotomy, laparoscopy allows the operator to complete the spleen hilum lymph node dissection under a clear field of view and helps to improve safety [56].

Cancer recurrence and the long-term survival rate are two critical outcomes for evaluating surgical interventions in oncological therapy. Based on the available data, postoperative cancer recurrence and the long-term survival rate in LG were similar to those in OG. Regarding the recurrence pattern, Song *et al*. [57] stated that the hematogenous pattern was most common after LG, followed by the locoregional pattern. This is consistent with the results of some of the included studies and other research [30]. The concern about dissemination of gastric cancer due to insufflated gas from pneumoperitoneum and port site or wound metastasis, although quite rare, has been emphasized. Port-site recurrence was seen in two of included studies [34,39]; however, it was not an event unique to LG, because there were also two cases of wound metastasis in OG group [39]. Zhao *et al*. and others [39,58] stated that laparoscopic surgery does not promote abdominal or trocar implantation of gastric cancer. As previously mentioned, researchers indicated that LG did not increase the risk of perigastric lymph node recurrence compared to OG [55]. Sato *et al*. [59] analyzed the difference between OG and LG in relation to D_1_, D_1+_ or D_2_ lymph node dissection using a hierarchical approach and found that the long-term results of LG were comparable to those of OG. Park *et al*. [55] analyzed the follow-up results of 239 cases of AGC treated with LG. The 5-year survival rates of T_2,_ T_3_ and T_4_ stage patients were 86.6%, 77.4% and 58.7%, respectively, which is similar to that for concurrent laparotomy [60,61].

However, there were several limitations that must be taken into account when considering the above-mentioned results: (1) tumor depth and nodal status were risk factors for recurrence, and survival for patients with pT_2_ cancer has been reported to be better than that for patients with other advanced stage [62,63]. Two of the included studies were limited to pT_2_ stage patients [32,41], and some of others mainly referred to stage IB-II or pT_2-3_ tumor invasion [34,36,38,40,42,43,45]. Hence, there should be an attitude of caution concerning laparoscopic resection of more advanced cases because relevant studies and clinical evidence are still deficient; (2) postoperative adjuvant chemotherapy has demonstrated a clear survival benefit compared to treatment with surgery alone [3,4]. However, some included studies failed to provide such information [31,33,37,38,40-42,44], which might have affected the results; (3) the homogeneity test for the continuous variables exhibited substantial heterogeneity due to the inherent flaws of a retrospective study, the uneven surgical skills of the different surgeons as well as regional differences, etc.

## Conclusions

The existing research shows that LG for AGC is safe and feasible, characterized by such advantages as less pain, fewer postoperative complications and rapid recovery. Moreover, our results suggest that the application of LG to this group results in adequate lymphadenectomy and similar recurrence and survival rates as OG. However, there were several limitations in this research. Therefore, the results mentioned above should be subject to verification by strictly designed, large-sample, multicenter RCTs with extended follow-up outcomes.

## Abbreviations

LG: Laparoscopic gastrectomy; AGC: Advanced gastric cancer; RR: Risk ratio; WMD: Weighted mean differences; CI: Confidence intervals; OG: Open gastrectomy; EGC: Early gastric cancer; RCT: Randomized controlled trial; NOS: Newcastle-Ottawa quality assessment scale; SD: Standard deviation; DFS: Disease-free survival; OS: Overall survival.

## Competing interests

The authors declare that they have no competing interests.

## Authors’ contributions

CK and MYP designed the study; XXW and PY performed the research and retrieved data; ZYC, ZRC and WD collected the data; CK wrote the article; MYP proofread and revised the manuscript. All authors read and approved the final manuscript.
